# Alterations in Degree Centrality and Functional Connectivity in Parkinson’s Disease Patients With Freezing of Gait: A Resting-State Functional Magnetic Resonance Imaging Study

**DOI:** 10.3389/fnins.2020.582079

**Published:** 2020-11-03

**Authors:** MiaoRan Guo, Yan Ren, HongMei Yu, HuaGuang Yang, ChengHao Cao, YingMei Li, GuoGuang Fan

**Affiliations:** ^1^Department of Radiology, The First Affiliated Hospital of China Medical University, Shenyang, China; ^2^Department of Neurology, The First Affiliated Hospital of China Medical University, Shenyang, China

**Keywords:** Parkinson’s disease, freezing of gait, degree centrality, functional connectivity, Resting-state fMRI

## Abstract

**Objective:**

Freezing of gait (FOG) is a common disabling motor symptom in Parkinson’s disease (PD), but the potential pathogenic mechanisms are still unclear.

**Methods:**

A total of 22 patients with PD with FOG (PD-FOG), 28 patients with PD without FOG (PD-nFOG), and 33 healthy controls (HCs) were recruited in this study. Degree centrality (DC)—a graph theory-based measurement of global connectivity at the voxel level by measuring the number of instantaneous functional connections between one region and the rest of the brain—can map brain hubs with high sensitivity, specificity, and reproducibility. DC was used to explore alterations in the centrality of PD-FOG correlated with brain node levels. PD-FOG cognitive network dysfunction was further revealed via a seed-based functional connectivity (FC) analysis. In addition, correlation analyses were carried out between clinical symptoms and acquired connectivity measurement.

**Results:**

Compared to the PD-nFOG group, the PD-FOG group showed remarkably increased DC values in the right middle frontal gyrus (RMFG). There were no significant differences in other gray matter regions. Importantly, the clinical severity of FOG was related to the mean DC values in the RMFG. This brain region served as a seed in secondary seed-based FC analysis, and we further found FC changes in the right precuneus, right inferior frontal gyrus, right superior frontal gyrus (SFG), and cerebellum.

**Conclusion:**

Increased RMFG activity and FC network alterations in the middle frontal cortex with the precuneus, inferior, and SFG, and the cerebellum may have great potential in brain dysfunction in PD with FOG.

## Introduction

Parkinson’s disease (PD) is a chronic progressive neurodegenerative disorder mainly characterized by motor impairments (Kalia and Lang, 2015). Notably, freezing of gait (FOG) is one of the most disabling motor symptoms, defined by “brief, episodic absence or marked reduction of forward progression of the feet despite the intention to walk” (Nutt et al., 2011). Nearly 50–80% of PD patients experience FOG (Macht et al., 2007), which contributes to falls and subsequent fractures or other complications, thereby seriously impacting patients’ quality of life (Snijders et al., 2011). Despite being a relatively common occurrence in PD, the underlying pathophysiological mechanisms of PD-FOG remain largely unclear.

In recent years, a growing body of neuroimaging studies have focused on exploring the imaging biomarkers of PD-FOG by using different methods. A structural magnetic resonance imaging (sMRI) study involving voxel-based morphometry (VBM) found widespread gray matter (GM) volume atrophy in the frontal–parietal cortical areas and cerebellum in PD-FOG (Jha et al., 2015). Similarly, one surface-based morphometry (SBM) study based on 21 patients with PD-FOG, 28 PD patients without FOG (PD-nFOG), and 19 healthy controls (HCs) revealed bilateral frontal, parietal, and occipital cortical thickness reductions in PD-FOG, which were significant in the middle frontal cortex (Pietracupa et al., 2018). The middle frontal gyrus is mainly involved in higher cognitive functions, especially executive functions (John et al., 2006). Furthermore, a functional MRI (fMRI) study using amplitude of low-frequency fluctuation (ALFF) also found abnormal ALFF in the frontal, parietal, and temporal areas and cerebellum in PD-FOG, and found a significant correlation between ALFF changes in the middle frontal gyrus and FOG severity (Mi et al., 2017). This finding suggested that executive function impairment in the frontal regions was involved in PD-FOG. Notably, most of the published neuroimaging studies of PD-FOG have mainly focused on structural abnormalities. To our knowledge, studies based on the resting state voxel-level whole-brain impaired neural networks are rare in literature, which has limited our understanding of PD-FOG.

Degree centrality (DC)—a powerful method to explore whole-brain neural network abnormalities—has recently gained more attention. It is based on graph theory and may reduce the possible bias caused by selecting brain regions according to the priori assumption (Buckner et al., 2009; Zuo et al., 2012). DC can reflect the relative importance of a node in a network (Yang et al., 2014), which has been widely used to study neurological and psychiatric disorders. Recently, one study used DC to explore cognitive dysfunction in patients with early bipolar disorder (Deng et al., 2019). Guo et al. (2016) revealed DC changes in the right middle frontal, precentral, and postcentral gyri, which fits a network dysfunction model in Alzheimer’s disease (AD). Yang et al. (2020) combined DC and functional connectivity (FC) to derive that the dorsolateral prefrontal cortex (DLPFC) may play a key role in multiple system atrophy (MSA) with cognitive impairment. All the above findings indicate that DC is a feasible method to explore resting state whole-brain neural network impairment. Furthermore, we used the regions that showed significant alterations in DC, combined with secondary seed-based FC analysis to provide insights into intra- and interregional neural network connectivity abnormalities in PD patients with FOG.

Patients with PD-FOG are observed to have impaired regions mainly concentrated in the frontal area and the cerebellum (Jha et al., 2015; Mi et al., 2017). Therefore, it is hypothesized that the brain functional impairments, particularly in the frontal executive and cerebellar networks, may be involved in the pathophysiology of PD-FOG. To test this hypothesis, we explored the specific region centrality alterations in PD-FOG patients through a voxel-based analysis of DC. In addition, we further conducted secondary seed-based FC analysis using the regions that showed significant alterations in DC as seeds. Finally, we investigated possible correlations between alterations of cerebral connectivity and the severity of FOG.

## Patients and Methods

### Participants

A total of 50 patients with idiopathic PD including 22 PD patients with FOG (9 females, 13 males; PD-FOG group) and 28 PD patients without FOG (12 females, 16 males, PD-nFOG group) diagnosed by two experienced neurologists using the Movement Disorder Society (MDS) clinical diagnostic criteria for PD (Postuma et al., 2015) were recruited from the Movement Disorder Specialist Department of Neurology at The First Affiliated Hospital of China Medical University from December 2016 to October 2019.

We also recruited 33 healthy controls (20 females, 13 males; HC group) matched by age, education, and sex from the same community as the patients by advertisement. We excluded atypical parkinsonism, as well as patients with depression, anxiety, visual disturbances, musculoskeletal disorders, or other mental disorders, to avoid a possible impact on FOG. Participants were also excluded if they had a history of alcoholism or any pathological findings on conventional MRI. The ethics committee of The First Affiliated Hospital of China Medical University approved this study. All participants provided written informed consent prior to undergoing magnetic resonance imaging (MRI).

### Diagnosis and Motor and Neuropsychological Assessment

Evaluation of motor disability and stage of PD included the Unified Parkinson’s Disease Rating Scale part III (UPDRS-III; Antonini et al., 2013) and the Hoehn and Yahr (2001) staging scale. The Mini-Mental State Examination (MMSE; Luria, 1975) was used to assess global cognitive function for each subject, and the Hamilton Depression Scale (HAMD; Hamilton, 1967) was used to assess mood and exclude emotional disorders.

The criteria for recruitment of PD-FOG patients were as follows: (1) a score of >0 on item 3 of the Freezing of Gait Questionnaire [FOGQ; “Do you feel that your feet get glued to the floor while walking, making a turn, or when trying to initiate walking (freezing)?”] (Nieuwboer et al., 2009) and (2) episodes of foot-movement cessation observed by two experienced neurologists when the patient performed a brief series of timed up-and-go trials, when required to make 180° turns to the left and right, or the patients’ verbal account of whether they had experienced this situation when the doctors imitated FOG.

### Rs-Functional Magnetic Resonance Imaging Image Acquisition and Preprocessing

All functional and structural MR images were acquired with a 3.0T MRI scanner (Magnetom Verio, Siemens, Erlangen, Germany) equipped with a 32-channel phased-array head coil at The First Affiliated Hospital of China Medical University. All patients were scanned following a 12-h period of medication withdrawal (off-state). Earplugs and foam pads were used to minimize machine noise and head motion. During scanning, all subjects were instructed to keep their eyes closed and stay quiet. Immediately after the scan, each patient was asked whether he or she fell asleep during the scan. The rs-fMRI scan used blood oxygen level-dependent (BOLD) single-shot echo-planar image (EPI) sequences with the following parameters: repetition time (TR), 2,500 ms; echo time (TE), 30 ms; flip angle, 90°; slice number, 43; slice thickness/gap, 3.5/0 mm; slice order, from 1, 3, 5–43 to 2, 4, 6–42, interleaved; field of view (FOV), 224 mm × 224 mm; matrix size, 64 × 64; and voxel size, 3.5 mm × 3.5 mm × 3.5 mm. High-resolution three-dimensional sagittal T1-weighted images were acquired in a magnetization-prepared rapid acquisition gradient echo (MPRAGE) sequence with the following parameters: TR, 5,000 ms; TE, 2,960 ms; flip angle, 12°; distance factor, 0.5; slice number, 176; slice thickness/gap, 1/0 mm; FOV, 256 mm × 256 mm; matrix size, 256 × 256; and voxel size, 1.0 mm × 1.0 mm × 1.0 mm.

Rs-fMRI data were preprocessed in MATLAB R2013b^1^ using SPM12^2^ and Data Processing and Analysis for (resting-state) Brain Imaging (DPABI) software (Yan et al., 2016) according to the standard procedure (Chao-Gan and Yu-Feng, 2010). The preprocessing steps were as follows. First, we removed the first 10 time-points to ensure a steady-state condition. The remaining images were obtained with slice timing and head motion correction. Any participants with head motion exceeding 1.5-mm maximum displacement in *x*, *y*, or *z* and/or 1.5° rotation during scans were excluded. We further calculated the mean framewise displacement (FD) values as a measure of the microscale head motion of each subject (Power et al., 2012); no participant was excluded in the present study. Several nuisance covariates including the Friston-24 parameter (six head motion parameters, six head motion parameters one time point before, and the 12 corresponding squared items; Friston et al., 1996), white matter signal, and cerebrospinal fluid signal were removed by linear regression. The remaining images were further filtered using a typical temporal bandpass (0.01–0.08 Hz) to reduce low-frequency drift and high-frequency physiological noise. Next, the generated data were realigned and spatially normalized to the standard Montreal Neurological Institute (MNI) template for intersubject comparison and then resampled to 3 mm × 3 mm × 3-mm isotropic voxels (Power et al., 2012). Finally, detrending was applied to remove the systematic drift of the baseline signal.

### Degree Centrality Analysis

Degree centrality is a graph theory-based approach to explore the degree of connection of a node in the network with all other nodes; furthermore, it enables whole-brain analysis at the voxel level, which reflects the FC within the brain network. Each voxel in the brain is regarded as a node with an edge indicating the FC of any two voxels (Zuo et al., 2012). Based on preprocessed data, voxel-wise DC value calculations were performed using the DPABI software. We extracted the BOLD time series of each voxel and computed Pearson’s correlation coefficients (r) between any pair of brain voxels within the whole-brain gray matter mask. Then, Pearson’s correlation data were normalized with Fisher’s *r*-to-*z* transformation to obtain the *Z*-score DC value map, and the whole-brain functional network was mapped with the threshold *r* > 0.25 in accordance with previous studies (Buckner et al., 2009; Li et al., 2016). After normalization, we smoothed the maps using a 6 mm × 6 mm × 6-mm full width at half maximum Gaussian kernel for further statistical analysis (data were preprocessed without smoothing). Owing to the uncertainty of interpretation, only positive Pearson correlation coefficients were considered in the DC calculations. To determine whether the main results depended on the choice of correlation thresholds, we applied another correlation threshold (*r* > 0.2) to recompute the DC maps and then reperformed statistical analysis (Supplementary Material 1).

### Functional Connectivity Analysis

To study the changes in resting state functional connectivity (rs-FC) in PD-FOG patients in detail, regions with significant group DC differences between the PD-FOG and PD-nFOG patients were used as seeds for further rs-FC. Seed regions were a sphere with a radius of 6 mm around the center voxels, and the reference time series for seeds were obtained by averaging the time series of all voxels within the seed region. Correlation analysis was then performed between the seeds and the remaining voxels. Finally, the correlation coefficients were converted into Fisher *z*-values to obtain a z-FC map for further statistical analysis. Six head motion parameters, global mean time courses, and white matter and cerebrospinal fluid (CSF) time courses were considered nuisance factors.

### Statistical Analysis

The demographic and clinical data were compared using SPSS 22.0 software, and the Kolmogorov–Smirnov test was applied to assess data normality. For the normally distributed variables, two-tailed independent-samples *t*-tests and analysis of variance (ANOVA) were used. Non-normally distributed data (education and MMSE scores) were evaluated using the Kruskal–Wallis *H* test. Chi-squared test was used to compare the sex distribution between groups. The significant level was set as *p* < 0.05.

Next, we performed one-way analysis of co-variance (ANCOVA) with age, sex, disease duration, and education as covariates to explore DC differences among the PD-FOG, PD-nFOG, and HC groups. Next, *post hoc* analysis was conducted with multiple comparison correction for ANCOVA (AlphaSim correction, *p* < 0.001). Then, two-sample *t*-tests were conducted to evaluate differences in DC between two patient groups, with age, sex, disease duration, and education as covariates. We performed a voxel-wise Pearson’s correlation analysis between the individual DC mapping and FOGQ scores to identify brain regions significantly associated with the severity of FOG in PD patients. We selected brain regions showing significant FOG severity-related areas in DC as a seed. We then conducted a secondary seed-based FC analysis to investigate FC network alterations in PD-FOG.

Additionally, we also added FD as a covariate to reperform the DC and FC analysis (Supplementary Material 2).

## Results

### Clinical Characteristics of Parkinson’s Disease and Healthy Control Groups

The clinical data for the PD and HC groups are presented in Table 1. No significant intergroup differences were observed with respect to age, sex, education, MMSE score, and FD values (all *p* > 0.05). There were no significant differences between the PD-FOG and PD-nFOG groups in terms of disease duration, UPDRS-III score, Hoehn and Yahr stage, HAMD score, and levodopa equivalent dose (all *p* > 0.05). As expected, the FOGQ score significantly differed between the two PD groups. The PD-FOG group had significantly higher FOGQ scores than the PD-nFOG group (*p* < 0.05).

**TABLE 1:** Demographic and clinical characteristics of all participants.

**Domain**	**Healthy controls (HCs; *n* = 33)**	**Parkinson’s disease without freezing of gait (PD-nFOG; *n* = 28)**	**PD with FOG (PD-FOG; *n* = 22)**	**F/χ2/Z**	***p*-Value**
Age (years)	63.06 ± 4.01	62.79 ± 6.22	61.32 ± 9.45	0.50	0.61
Sex (male/female)	13/20	16/12	13/9	2.77	0.25
Education (years)	12 (9, 16)	9 (3, 16)	9(6, 15)	2.14	0.34
Disease duration (years)	NA	5.04 ± 3.97	4.33 ± 3.20	0.46	0.50
LEED (mg/day)	NA	430.96 ± 88.23	479.45 ± 114.21	2.87	0.09
Unified Parkinson’s Disease Rating Scale part III (UPDRS-III)	NA	28.14 ± 17.57	28 ± 12.46	0.00	0.97
Hoehn and Yahr	NA	2.09 ± 0.61	1.96 ± 0.67	0.55	0.46
Hamilton Depression Scale (HAMD)	NA	10.43 ± 7.02	11.27 ± 9.23	0.14	0.72
Mini-Mental State Examination (MMSE) score	28(25, 30)	27(18, 30)	28(25, 30)	1.20	0.55
Freezing of Gait Questionnaire (FOGQ)	NA	1.21 ± 0.96	11.05 ± 4.72	116.25	0.00
Framewise displacement (FD; mm)	0.09 ± 0.01	0.12 ± 0.01	0.09 ± 0.00	1.50	0.23

*Values distributed normally or non-normally are presented as mean ± SD or median (minimum and maximum).*

### Degree Centrality Analysis

**FIGURE 1:**
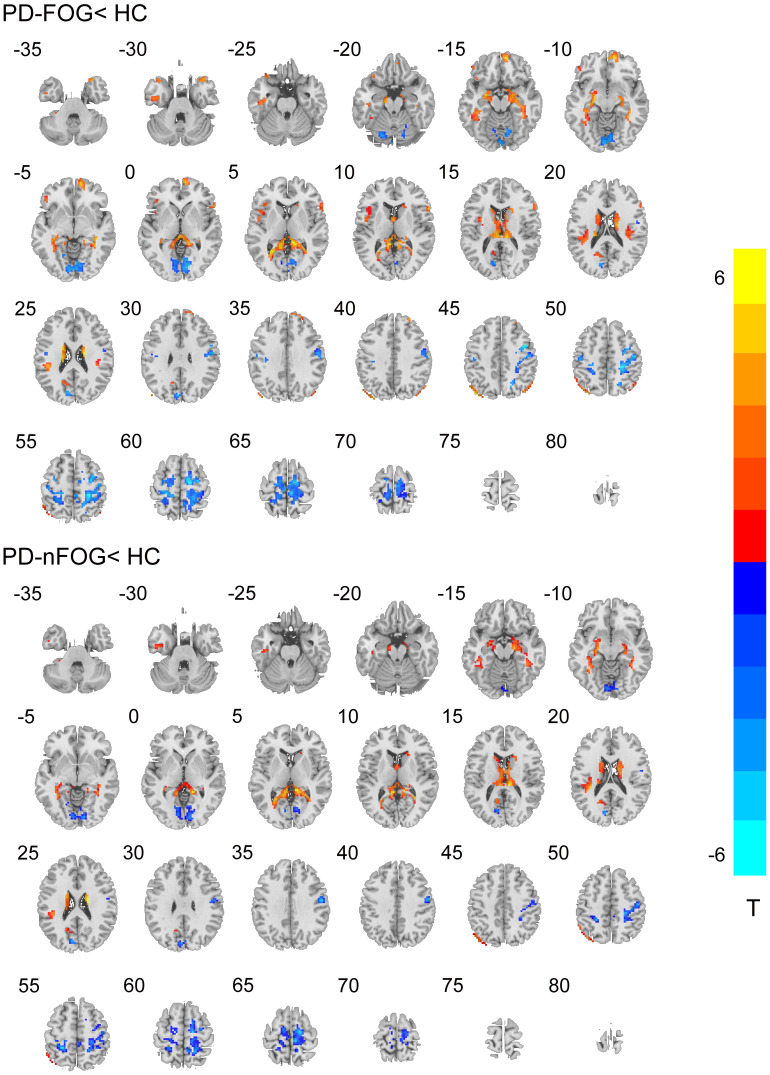
Degree centrality (DC) result maps of the comparison between patients and healthy control (HC) groups on axial images (*p* < 0.001, AlphaSim corrected). The left side of the image corresponds to the left side of the brain in axial orientation. Slice coordinates according to the Montreal Neurological Institute (MNI) space are shown on the upper left corner of the slices, indicating the *Z*-axis in axial orientation. The *t*-value scale is seen to the right of the image.

**FIGURE 2:**
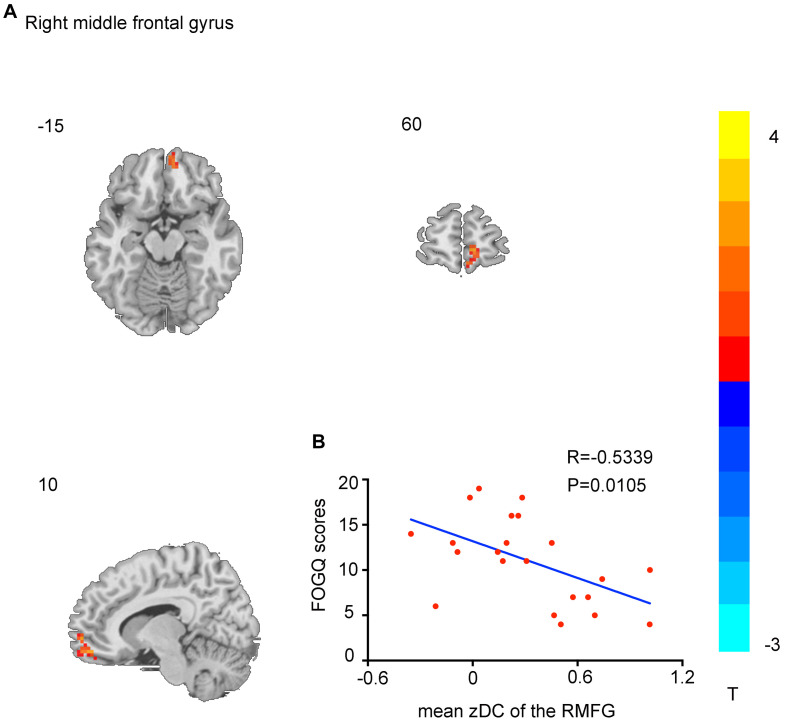
Brain regions showing DC differences between Parkinson’s disease with freezing of gait (PD-FOG) and PD patients without FOG (PD-nFOG) groups. Only right middle frontal gyrus (RMFG) had decreased DC (*p* < 0.001, AlphaSim-corrected). The *t*-value scale is seen to the right of the image **(A)**. The scatter plot shows the negative correlation between RMFG DC values and Freezing of Gait Questionnaire (FOGQ) scores in PD-FOG patients **(B)**.

Compared with the HC, individuals with PD showed significantly enhanced DC in the bilateral caudate and left inferior temporal gyrus and remarkably decreased DC in the bilateral precentral gyrus, bilateral postcentral gyrus, and bilateral cerebellum (AlphaSim correction, *p* < 0.001). Notably, direct comparison of PD-FOG and PD-nFOG groups demonstrated remarkable differences only in the right middle frontal gyrus (RMFG; Table 2 and Figures 1, 2A).

**TABLE 2:** Differences in the degree centrality (DC) values between PD patients and HCs.

**Brain regions**	**Cluster size**	**Brodmann area**	**Montreal neurological institute (MNI) coordinates**			***T*-value**
				
			***X***	***Y***	***Z***	
PD-FOG Vs HC						
Precentral_R	147	4/6	13	−20	65	−3.0256
Precentral_L	40	4/6	−10	−7	65	−3.8219
Postcentral_R	249	3	21	−44	63	−3.3354
Postcentral_L	102	3	−23	−45	59	−3.3178
Frontal_Mid_R	48	10	8	57	−13	6.8132
Frontal_Sup_R	68	12	12	64	3	4.9057
Frontal_Mid_L	56	10	−46	33	−6	3.9194
Caudate_R	57	NA	17	−7	20	5.3057
Caudate_L	65	NA	−8	−26	12	4.7739
Precuneus_L	42	27	−6	−48	13	3.7815
Precuneus_R	33	27	12	−51	10	4.0065
Temporal_Inf_L	84	20	−43	−34	24	5.0039
Cerebelum_L	78	NA	−20	−73	−20	−4.3242
Cerebelum_R	68	NA	14	−72	−16	−3.9551
PD-nFOG Vs HC						
Precentral_R	151	4/6	16	−14	66	−5.7593
Precentral_L	36	4/6	−12	−11	66	−4.6072
Postcentral_R	253	3	22	−46	56	−5.7651
Postcentral_L	99	3	−23	−43	55	−6.4512
Caudate_R	57	NA	16	−6	25	6.7735
Caudate_L	65	NA	−16	−1	24	6.2865
Temporal_Inf_L	87	20	−38	−34	21	4.8056
Cerebelum_L	80	NA	−18	−75	−21	−5.3674
Cerebelum_R	69	NA	17	−70	−19	−5.6143
PD-FOG Vs PD-nFOG						
Frontal_Mid_R	25	11/12	16	62	−9	3.3550

### Seed-Based rsFC Analysis

**FIGURE 3:**
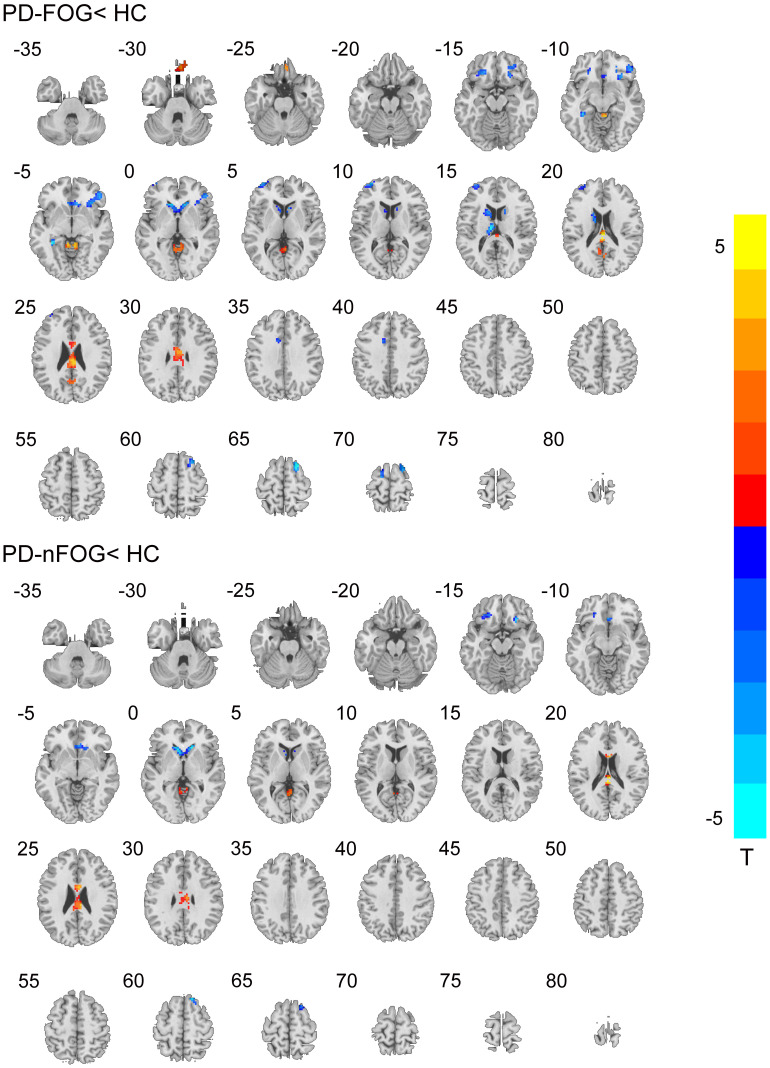
Functional connectivity (FC) from RMFG to the other brain regions. Red and blue indicate increased and decreased FC, respectively, in the PD-FOG and PD-nFOG groups when compared with HC. Differences were considered significant at *p* < 0.001 (AlphaSim-corrected).

**FIGURE 4:**
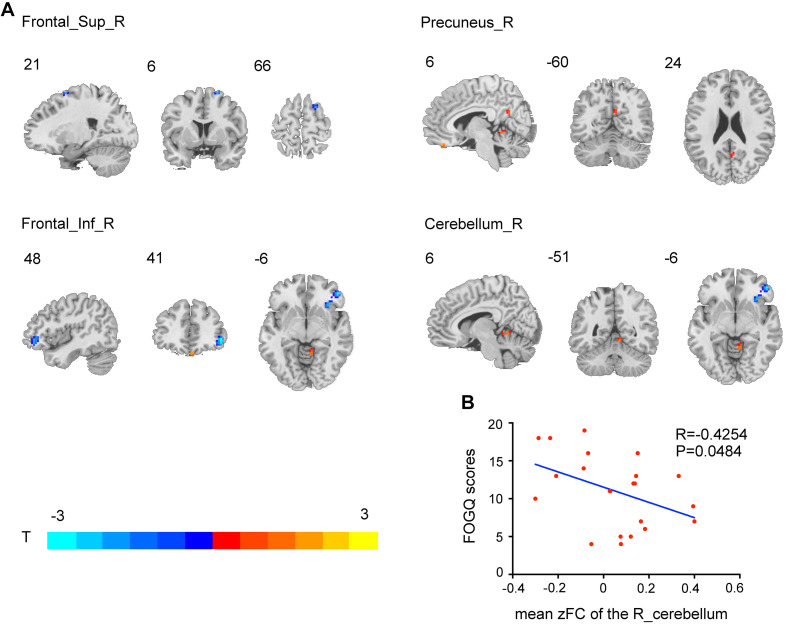
Brain regions showing RMFG-related FC alterations in the PD-FOG group compared with the PD-nFOG group (*p* < 0.001, AlphaSim-corrected). Red represents increased rsFC in the right precuneus and cerebellum, and blue represents decreased rsFC in the right inferior and superior frontal gyrus. The *t*-value scale is seen on the lower left of the image **(A)**. Correlations between FC and FOGQ scores. FOGQ scores were negatively correlated with impaired connectivity between the RMFG and right cerebellum in the PD-FOG group **(B)**.

Compared to HCs, the PD-FOG group showed RMFG seed-based FC impairment in the bilateral inferior frontal gyrus (IFG), middle frontal gyrus, superior frontal gyrus (SFG), left caudate, and cerebellum. PD-nFOG patients showed reduced RMFG-related FC in the left, right SFG, bilateral middle frontal gyrus, and bilateral caudate. In addition, PD-FOG patients had significantly lower RMFG seed-based FC in the right IFG and right SFG and higher FC alterations in the right precuneus and cerebellum than PD-nFOG patients (Table 3 and Figures 3, 4A).

**TABLE 3:** Differences in rs-functional connectivity (FC) between PD patients and HCs.

**Brain regions**	**Cluster size**	**Brodmann area**	**MNI coordinates**			***T*-value**
			***X***	***Y***	***Z***	
PD-FOG Vs HC						
Frontal_Inf_Orb_R	83	12	48	42	−3	−4.4847
Frontal_Inf_Orb_L	17	12	−33	60	9	−4.6182
Frontal_Mid_L	60	10	−18	33	−12	−4.7295
Frontal_Mid_R	14	10	20	31	−11	−4.3108
Frontal_Sup_R	46	11	22	12	66	−5.1094
Frontal_Sup_L	16	11	−33	57	9	−3.9982
Caudate_L	88	NA	−9	−15	15	−4.9720
Vermis	38	NA	6	−42	−6	5.2355
PD-nFOG Vs HC						
Frontal_Inf_Orb_L	19	12	−35	56	13	−4.6872
Frontal_Mid_L	66	10	−21	30	−12	−5.0237
Frontal_Mid_R	14	10	21	27	−12	−4.8675
Frontal_Sup_R	36	11	15	30	60	−5.1533
Caudate_L	107	NA	−13	2	18	−3.8801
Caudate_R	54	NA	17	7	19	−3.4976
PD-FOG Vs PD- nFOG						
Frontal_Inf_R	51	11	48	42	−6	−3.4653
Frontal_Sup_R	15	10	21	6	66	−2.7405
Cerebellum_R	13	NA	6	−51	−6	2.6312
Precuneus_R	8	31	6	−60	24	2.4746

### Correlation Analysis

Figure 2B shows the correlation analysis between the individual DC mapping and FOGQ scores of patients with PD (*p* < 0.001, AlphaSim-corrected for multiple comparisons, cluster size > 459 mm^3^). The RMFG-dependent DC exhibited a notably negative correlation with FOGQ scores. Scatter plots of the correlation coefficient are shown in Figure 2B.

In addition, correlation analysis was conducted between the individual RMFG-related FC network alterations and FOGQ scores in PD-FOG patients, and the results indicated a negative correlation between the FOGQ scores and mean RMFG-related FC values. Scatter plots of the correlation coefficient are shown in Figure 4B.

## Discussion

Freezing of gait in patients with PD is a distinct gait abnormality, the underlying mechanism of which is still unclear. In this study, we combined DC and secondary seed-based FC to evaluate specific regional differences in DC and related whole-brain network FC abnormalities among PD-FOG patients, PD-nFOG patients, and HCs. Patients with PD-FOG showed more extensive area changes to brain regions, mainly manifesting as increased DC values in the RMFG, than PD-nFOG patients. Moreover, the increased DC values in RMFG were significantly negatively correlated with FOGQ scores in PD patients. Second, compared to HCs, patients with PD-FOG shared similar abnormal DC areas with PD-nFOG patients in the bilateral caudate, bilateral pre- and postcentral gyrus, bilateral cerebellum, and left inferior temporal gyrus. Finally, compared to the PD-nFOG group, the PD-FOG group showed increased FC in the RMFG precuneus and cerebellum and decreased FC in the RMFG-IFG and SFG.

Notably, patients with PD-FOG showed a higher DC value in the RMFG than the PD-nFOG patients. Moreover, DC values in the RMFG were significantly correlated with FOGQ scores, which suggested that the MFG may play a crucial role in the mechanism of FOG in PD patients. The MFG is located between the SFG and IFG and is the major component of the DLPFC (John et al., 2006). As a key node of the cognitive control network, the DLPFC mediates more advanced functions such as executive attention, motor planning, and decision making (Lau et al., 2019). This role suggested that, in addition to being a motor function impairment, FOG may also be related to cognitive and executive functional impairment in PD patients. Consistent with our study, Brugger et al. (2015) used VBM and found GM atrophy in the prefrontal cortex in PD-FOG and a correlation between the degree of GM atrophy and FOG severity. Patients with PD-FOG have significant cognitive dysfunction with frontal lobe executive impairment. Furthermore, positron emission tomography (PET)- (Tard et al., 2015; Gallardo et al., 2018) and single photon emission computed tomography (SPECT)-based studies (Imamura et al., 2012) showed that PD-FOG patients have significantly reduced metabolism or perfusion in the frontal areas. In addition, Chawla et al. suggested that FOG also involved the perception–cognition system that is mediated mainly by the frontal cortex. PD patients may recruit attention and cognition resources to compensate for the impairment in motor function to achieve a more normal gait, revealing that cognition-related damage to the frontal cortex is correlated with FOG (Chawla et al., 2014). Taken together, our findings reinforced that frontal gyrus dysfunction, especially in the MFG, is a key cortical pathogenic hub in PD-FOG patients.

In fact, normal walking is not only an automatic movement that includes stepping and balance but also requires the integration of balance among attention, execution, visual movement networks, afferent information processing, and intentional adjustments (Snijders et al., 2007; Yogev-Seligmann et al., 2008; Al-Yahya et al., 2011). Therefore, FOG may exist as a motor manifestation of a global dysfunction in the concurrent processing of information across the neuronal network (Shine et al., 2013). Converging evidence suggests that the prefrontal cortex (PFC) may play a crucial role in controlling gait patterns when environmental conditions change (Nutt et al., 1993). Therefore, the PFC network dysfunction affects its corresponding executive functions (such as starting, walking, or turning), resulting in lack of instructions related to gait and further leading to FOG. Notably, DC represents the relative importance of a node in a network (Yang et al., 2014). We also found that the increased DC values in the RMFG were negatively correlated with FOGQ. PD-FOG is reportedly related to the functional decoupling between the cognitive control network and basal ganglia (Shine et al., 2013). We speculated that the increased DC in the RMFG may play a compensation mechanism of the cognitive control function in PD-FOG. However, disease duration and other factors can produce different results, and further research is required to elucidate its compensatory or pathological mechanisms.

To further investigate whole-brain functional network alterations in patients with PD-FOG, we used RMFG as a seed combined with a secondary FC and found altered RMFG-FC in the frontal–parietal areas, including the right IFG, SFG, and precuneus, compared with the PD-nFOG group. The IFG and SFG are both part of the prefrontal cortex, adjacent to the MFG, and control advanced cognitive functions. Moreover, the precuneus is located on the superior parietal lobule on the medial surface of the brain hemisphere and is involved in visuospatial processing, episodic memory, self-reflection, and consciousness (Hebscher et al., 2019). One study found that compared to controls, PD-FOG patients have reduced precuneus cortical thickness (Pietracupa et al., 2018). A recent study also showed that the chief components of the default mode network (DMN) are the prefrontal cortex, ventral anterior cingulate cortex, posterior cingulate cortex, precuneus, medial parietal cortex, and inferior parietal cortex. Notably, the PFC and precuneus both represent critical nodes in the DMN, suggesting that FOG may involve DMN alterations. The characteristics of the DMN include ongoing intrinsic brain activity during rest and deactivation during tasks, but it also exhibits task-related increases independently or in multiple components in the network (Krajcovicova et al., 2012). Furthermore, the network is believed to be involved in higher-order cognition, such as self-referential introspective, autobiographical memory retrieval, monitoring of surrounding environment, and anticipating the future (Raichle and Snyder, 2007; Tessitore et al., 2012; Raichle, 2015). DMN dysfunction is found widely in different neurodegenerative disorders including AD (Zheng et al., 2017; Liu et al., 2018), MSA (Yang et al., 2020), and schizophrenia (Assaf et al., 2010), and PD (Chen et al., 2017). A recent study uncovered a link between lower DMN activity and PD-FOG (Canu et al., 2015), and another study suggested that DMN alterations are correlated with cognitive impairment (Grieder et al., 2018). We also found in the current study that the RMFG-FC of the right SFG and IFG showed decreased changes in PD-FOG, suggesting that their activity was reduced, reflecting the presence of pathological damage in the DMN. The RMFG-FC of the right precuneus showed increased alterations, suggesting that local brain activity was enhanced in the DMN and reflected the compensatory function of DMN. In brief, our results further revealed that interruption of the dynamic equilibrium between the MFG and DMN may reduce the ability of the cognitive system to prepare for future task execution in PD-FOG patients.

We also found extensive cerebellar DC abnormalities in both PD-FOG and PD-nFOG patients compared to HCs. Previous studies (Timmermann et al., 2003; Dirkx et al., 2017) have shown that the cerebellum takes part in motor symptoms in PD patients, especially in the resting tremor. In fact, the cerebellum is thought to be involved in multiple locomotive functions, including internal postural models, perception of body motion, motor planning, and movement adaptation to environmental changes (Middleton and Strick, 2000; Bostan et al., 2013). Moreover, the cerebellar locomotor region has been proposed to be a pacemaker, providing rhythmic output to control temporal components of gait (Fling et al., 2013; Zwergal et al., 2013). It is also important for regulating ongoing movement and maintaining stable standing posture (Takakusaki, 2008). However, compared with PD-nFOG, PD-FOG showed that an increased FC was observed between the RMFG with the right cerebellum. As mentioned before, the RMFG belongs to the frontal region, which plays a key role in the cognitive control network. The synergistic effect of the cerebellum with the frontal network has indicated that aside from its traditional integration of motor function, the cerebellum also participates in the regulation of non-motor functions such as cognition. Yang et al. (2019) reported that cerebellar abnormalities in patients with MSA are related to the cognitive impairment process. Wang et al. (2018) suggested that the cerebellum plays a key role in emotion regulation in PD patients with depression. Similarly, some neuroimaging studies have found an abnormal cortico-pontine-cerebello-thalamo-cortical pathway (Gilman et al., 2010; Schweder et al., 2010) and abnormal functional activation of the cerebellum in PD-FOG (Ballanger et al., 2008; Palmer et al., 2009). Consistent with previous studies, our results indicate that the cerebellum mediates movement and subserves cognitive function related to the frontal network. PD-FOG patients showed increased RMFG-FC in the right cerebellum and a negative correlation with FOGQ scores, which may suggest a cerebellar compensatory effect in PD-FOG. Paradoxically, one study suggested that the altered cerebellum activity may be a pathophysiological mechanism in PD-FOG (Bharti et al., 2019). Taken together, we speculated that cerebellar function is dynamic and continuous in the process of PD, which increases spontaneous activity or FC with attempts to compensate gait in early-stage PD, but with pathological progress, compensation gradually enters the stage of decompensation. The PD patients we enrolled had a relatively short course of disease (mean, 4.7 years), and the cerebellum continued to play a compensatory role despite impairment. However, future longitudinal studies are required to confirm this preliminary conclusion.

We also found that compared with HCs, PD patients showed DC changes in the bilateral central anterior gyrus, central posterior gyrus, caudate, and cerebellum and altered caudate RMFG-FC. However, the PD-FOG group showed more extensive changes with increased DC in RMFG and RMFG-DMN and RMFG-cerebellum network alterations than the PD-nFOG group. A previous study showed that the basal ganglia may play an important role in PD-FOG (Lewis and Barker, 2009). Similarly, a study using VBM found that caudate volume was related to the severity of FOG (Herman et al., 2014). However, we failed to find significant basal ganglia differences between the PD-FOG and PD-nFOG groups, which may relate to the sample size or disease duration. Further long-term observations and research are needed. Additionally, compared with PD-nFOG, PD-FOG patients showed increased DC only in the RMFG. Recently, Pietracupa et al. (2018) reported a predominant impairment in white matter bundles in the right hemisphere in PD-FOG patients. Similarly, most studies have shown hypometabolism in the frontal and parietal regions in PD-FOG primarily involving the right hemisphere (Bartels et al., 2006; Gallardo et al., 2018). Our results were in agreement with a growing body of literature, which have reported that the right hemispheric circuitry of the brain appears to be selectively affected in PD-FOG. The brain is organized with certain specialized functions lateralized to each hemisphere. For example, the left hemisphere is preferentially involved in verbal processing and motor control, whereas the right hemisphere plays a stronger role in spatial cognition, body schema, and action inhibition (Fling et al., 2013). Other studies have shown that primarily left-sided symptoms in PD (right hemisphere) are associated with slower gait and poorer judgment of narrow doorways (Lee et al., 2001; van der Hoorn et al., 2012). We performed a statistical analysis of the dominant side of the symptom, and there was no significant difference between the two patient groups (*p* = 0.63, *p* > 0.05); thus, this is inadequate to explain the relationship between the dominant side of the symptoms and brain network alterations. Therefore, future studies should clarify this laterality.

Our study has some limitations. First, FOG is episodic and unpredictable, often not appearing during evaluations. We depended on patients’ self-reported FOG rather than being objectively measured; thus, the severity of FOG measure might have been biased. Second, our sample size was relatively small, reducing the power of the statistical analysis, which may increase the likelihood of false-negative results. Therefore, to ensure the reliability of our findings, we plan to enroll more subjects for a better understanding of the neuroimaging features of PD patients with FOG.

## Conclusion

We combined DC and a secondary, seed-based FC approach derived from rs-fMRI to explore whole-brain FC in PD-FOG. We found that PD-FOG is associated with the RMFG and RMFG-related rsFC abnormalities, mainly in the DMN (right prefrontal cortex and right precuneus) and cerebellum. These regions likely play a key role as pathogenesis hubs in PD-FOG. Overall, we believe our findings contribute to new insights into the neural mechanisms underlying the development of FOG in PD.

## Data Availability Statement

The raw data supporting the conclusions of this article will be made available by the authors, without undue reservation.

## Ethics Statement

The studies involving human participants were reviewed and approved by Ethics Committee of The First Affiliated Hospital of China Medical University. The patients/participants provided their written informed consent to participate in this study.

## Author Contributions

MG and GF conceived the study, participated in its design, and wrote the manuscript. YR and HMY revised important intellectual content. HGY, CC, and YL performed acquisition of data. MG analyzed and interpreted the data. All authors read and approved the final manuscript.

## Conflict of Interest

The authors declare that the research was conducted in the absence of any commercial or financial relationships that could be construed as a potential conflict of interest.

## Footnotes

1 https://www.mathworks.com

2 http://www.fil.ion.ucl.ac.uk/spm
