# Supplementary figures and images for: Alterations in Degree Centrality and Functional Connectivity in Parkinson’s Disease Patients With Freezing of Gait: A Resting-State Functional Magnetic Resonance Imaging Study
Source: Front Neurosci. 2020 Nov 3;14:582079. doi: 10.3389/fnins.2020.582079 (PMC7670067; doi:10.3389/fnins.2020.582079)

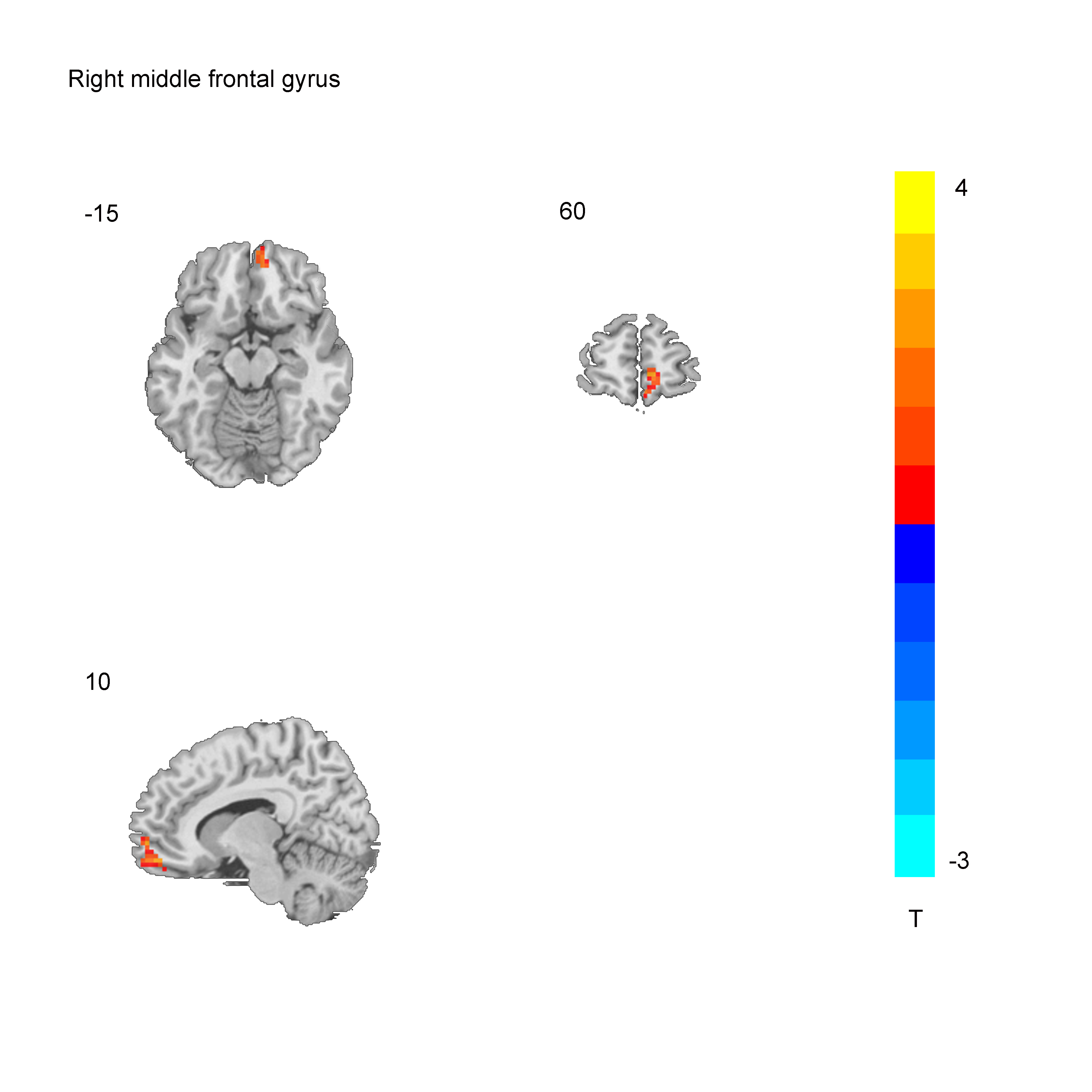

Supplement: Supplementary file 2 [file Image_2.JPEG]

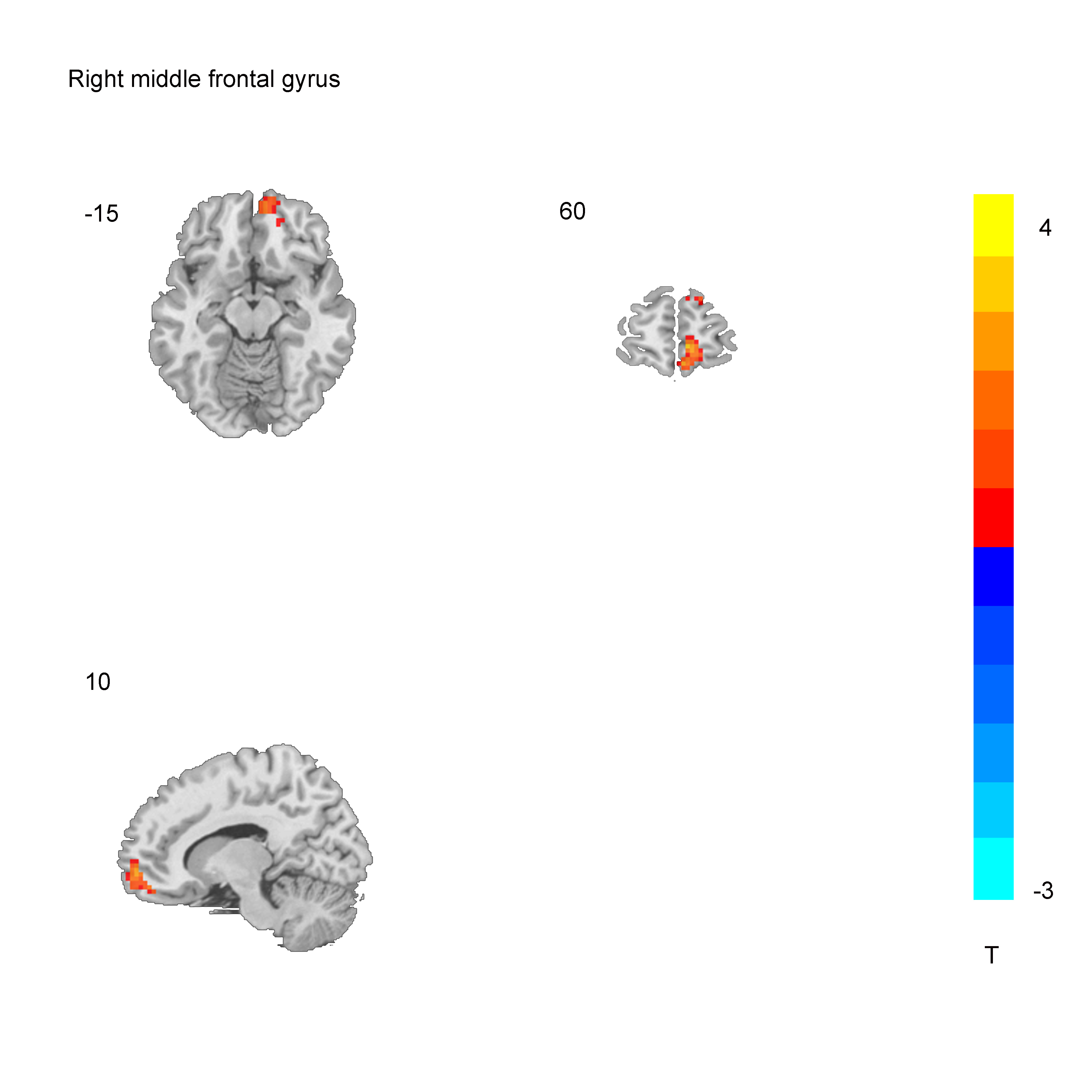

Supplement: Supplementary file 4 [file Image_4.JPEG]

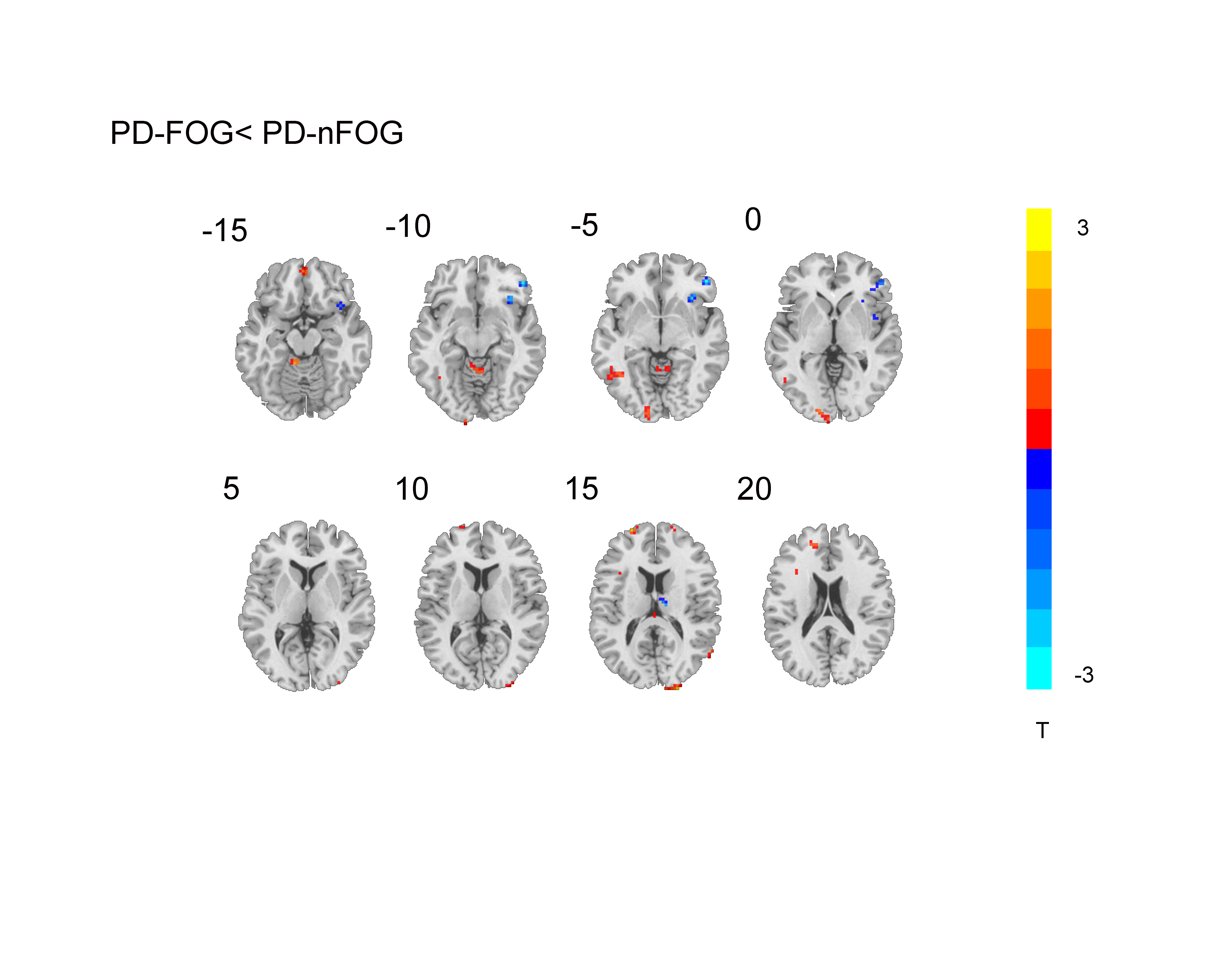

Supplement: Supplementary file 6 [file Image_6.JPEG]

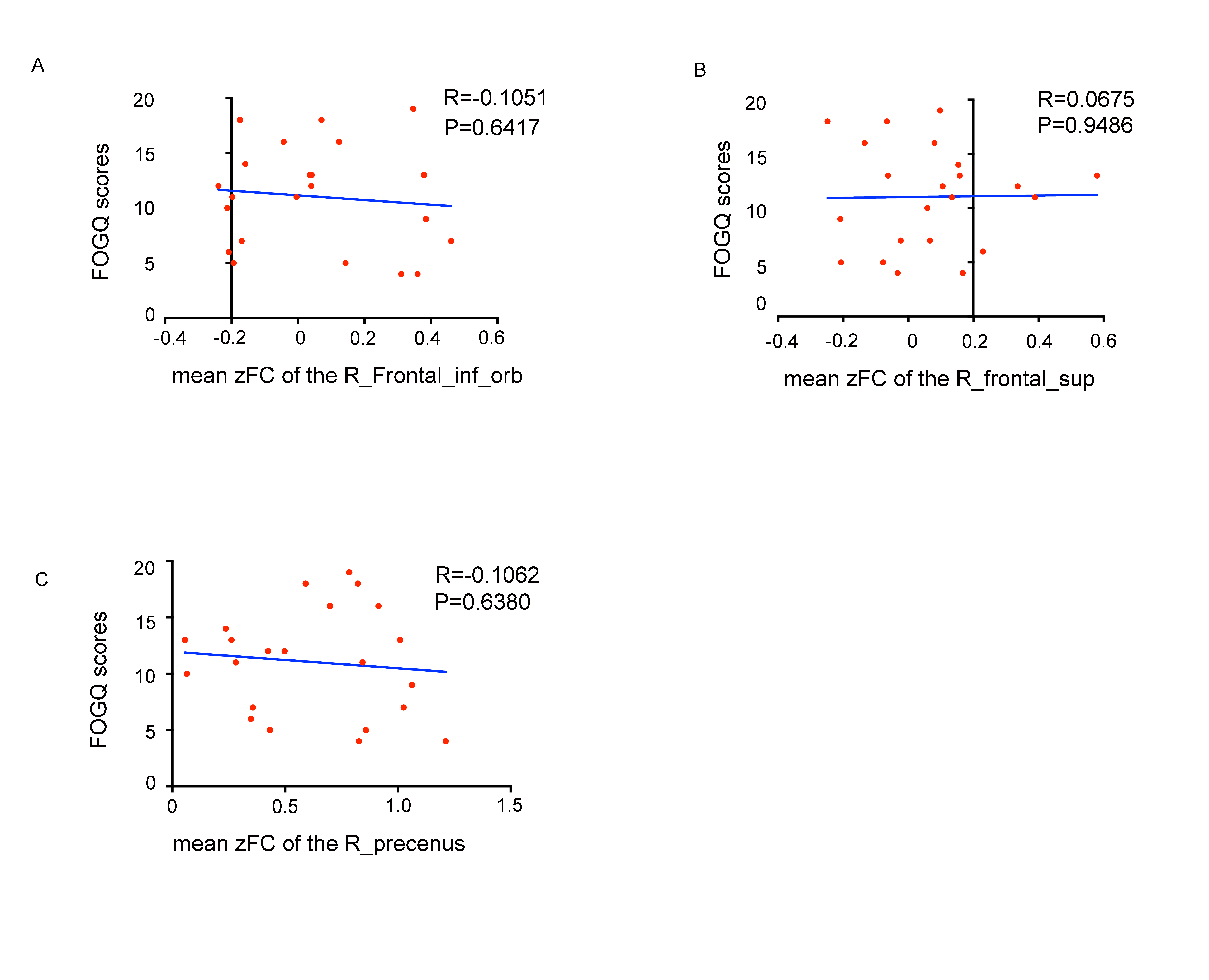

Supplement: Supplementary file 7 [file Image_7.JPEG]
