# Supplementary material for: Alterations in Degree Centrality and Functional Connectivity in Parkinson’s Disease Patients With Freezing of Gait: A Resting-State Functional Magnetic Resonance Imaging Study
Source: Front Neurosci. 2020 Nov 3;14:582079. doi: 10.3389/fnins.2020.582079 (PMC7670067; doi:10.3389/fnins.2020.582079)
Supplement: Supplementary file 8 [file Table_1.DOCX]

**FIGURE S1.1**. DC result maps of the comparison between patients and HC groups on axial images (r>0.2) (p < 0.001, AlphaSim corrected).

**FIGURE S1.2.** Brain regions showing DC differences between PD-FOG and PD-nFOG groups. Only RMFG had decreased DC (r>0.2) (P < 0.001, AlphaSim-corrected).

**FIGURE S2.1**. DC result maps of the comparison between patients and HC groups on axial images (p < 0.001, AlphaSim corrected). Add FD value as a covariate.

**FIGURE S2.2.** Brain regions showing DC differences between PD-FOG and PD-nFOG groups. Only RMFG had decreased DC (P < 0.001, AlphaSim-corrected). Add FD value as a covariate.

**FIGURE S2.3.** FC from the RMFG to the other brain regions. Red and blue indicate increased and decreased FC, respectively, in the PD-FOG and PD-nFOG groups when compared with HC. Differences were considered significant at (p < 0.001, AlphaSim-corrected). Add FD value as a covariate.

**FIGURE S2.4.** Brain regions showing RMFG-related FC alterations in the PD-FOG group compared with the PD-nFOG group (P < 0.001, AlphaSim-corrected). Red represents increased rsFC in the right precuneus and cerebellum, and blue represents decreased rsFC in the right inferior and superior frontal gyrus. Add FD value as a covariate.

**FIGURE S3.** Correlations between FC and FOGQ scores.
